# Doses of fluoride toothpaste for children up to 24 months

**DOI:** 10.1038/s41405-024-00187-7

**Published:** 2024-01-31

**Authors:** Henny Sudradjat, Frederic Meyer, Pascal Fandrich, Erik Schulze zur Wiesche, Hardy Limeback, Joachim Enax

**Affiliations:** 1 Dr. Kurt Wolff GmbH & Co. KG, Research Department, Johanneswerkstr. 34-36, 33611 Bielefeld, Germany; 2 Private dental practice, Braunschweig, Germany; 3 Faculty of Dentistry, University of Toronto, Toronto, ON M5G 1G6 Canada

**Keywords:** Dentistry, Dental public health

## Abstract

**Aim:**

The aim of this study was to test the dose of fluoride toothpaste by parents for their children aged up to 24 months.

**Methods:**

Parents who use fluoride toothpastes for their children were asked to dose two commercially available toothpastes (A and B) with 1000 ppm fluoride each for their children as they would normally do at home. The toothpaste amounts were weighed, and as reference, the weight of an ‘optimal’ grain of rice-size amount of each toothpaste was used.

**Results:**

61 parents dosed a mean of 0.263 ± 0.172 g toothpaste A and 0.281 ± 0.145 g toothpaste B. The parents’ mean doses were 5.9 times higher for toothpaste A and 7.2 times higher for toothpaste B than an ‘optimal’ grain of rice-size amount (the reference dose as recommended). The difference between parent’s and reference dose was statistically significant (*p* < 0.001). Moreover, 39.3% of parents were not aware about conditions of use and warnings that have to be printed on the package of fluoride toothpastes.

**Conclusion:**

In this study, parents significantly overdosed the toothpaste for their children. To avoid fluoride intake from toothpaste, parents can choose fluoride-free alternatives for the oral care of their infants and toddlers.

## Introduction

Daily use of toothpaste and toothbrush is crucial to prevent early childhood caries (ECC) [1–3]. Toothpastes contain many different ingredients including abrasives, surfactants, agents used for caries protection and remineralization, and antibacterial agents [3, 4]. Fluoride is used around the globe as anti-caries agent in toothpastes in various forms such as sodium fluoride (NaF), stannous fluoride (SnF_2_), or sodium monofluorophosphate (Na_2_PO_3_F) [3].

Most toothpastes in the European Union are classified as cosmetic products. According to regulation (EC) No 1223/2009 of the *European Parliament* and of the *Council* of 30 November 2009 on cosmetic products special conditions of use and warnings are mandatory for fluoride toothpastes [5]: “For any toothpaste containing 0.1 to 0.15% fluoride unless it is already labeled as contra-indicated for children (e.g., ‘for adult use only’) the following labeling is obligatory”: ‘Children of 6 years and younger: Use a pea sized amount for supervised brushing to minimize swallowing. In case of intake of fluoride from other sources consult a dentist or doctor’” [5].

According to the *European Academy of Paediatric Dentistry*, children’s toothpaste with fluoride have to be dosed in small amounts, i.e., a grain of rice-size or pea-size amount of toothpaste should be dosed, depending on the age of the child (Table 1) [6]. This is also recommended in Germany [7]. Instead of using a rice-size amount of fluoride toothpaste for young children, a recommendation to use of a smear-size amount of fluoride toothpaste is sometimes made (e.g., in North America) [8].

**Table 1:** Recommendations of the *European Academy of Paediatric Dentistry* on the dose of fluoride toothpaste for children, taken from [6].

Age	Toothpaste amount	Fluoride concentration (ppm)
From the first tooth up to 24 months	Grain of rice	1000
2–6 years	Pea	1000
> 6 years	Up to full length of brush	1450

The use of small toothpaste amounts (i.e., grain of rice and pea) came about as a result of concerns of toxicological effects of fluoride, especially for infants and toddlers [9, 10]. Although fluorides reduce the risk of caries [11], fluoridated oral care products for infants and children have to be used with caution as there is a constantly increasing number of studies that have shown negative effects of fluoride on the human body, e.g., there are many recent review papers demonstrating concerns regarding chronic toxicity of fluoride (e.g., through fluoride from drinking water) and more research is needed to also analyze potential negative chronic effects from fluoride intake from toothpastes on children’s health [10, 12–14].

There are different sources of fluoride [15, 16]. The main sources of fluoride for infants and toddlers include fluoride toothpastes, fluoride tablets, fluoridated salt, and certain infants formulas based on soy [17]. In some countries the drinking water is artificially fluoridated or the ground water contains naturally high fluoride amounts [15, 18–20]. Artificially fluoridate drinking water in the U.S. contains approximately 0.7 mg/L fluoride [20]. There are also various sources of fluoride in food and beverages (Table 2). Besides fluoride toothpastes and artificially fluoridated drinking water there are other non-natural fluoride sources, e.g., fluoride tablets (with vitamin D) which usually contain 0.25 mg fluoride per tablet [7], and fluoridated salt which can contain up to 310 mg/kg fluoride [21]. Also, professionally applied fluoride varnishes are used in children usually twice a year (e.g., with 5% sodium fluoride) [22].

**Table 2:** Sources of fluoride in food and beverages (examples).

Source	Fluoride concentration	Reference
Soybean beverages	8.5–15.5 mg/L	[51]
Black tea	1.6–6.1 mg/L	[52]
Rice	0.53–3.61 mg/kg	[16]
Bananas	0.86–1.98 mg/kg	[16]
Coffee	0.845–1.465 mg/L	[53]
Cow’s milk	0.016–0.18 mg/L	[54]

As described above, fluorides are used in various forms. However, and despite of the frequent use of fluorides, the prevalence of ECC is still very high around the globe. The overall global pooled prevalence of ECC was reported in a recent systematic review to be 48% [95% CI: 43; 53], i.e. Africa 30% [19; 45], Americas 48% [42; 54], Asia 52% [43; 61], Europe 43% [24;66], Oceania 82% [73; 89] [23]. Moreover, a global trend towards a significant lower prevalence of ECC could not been observed, i.e., the prevalence of ECC was 55% [31,76] in the 1990s, 45% [37,53] in the 2000s, and 49% [42,55] in the 2010s [23].

For children aged 18–30 months it was reported that 64.3–83.9% (mean) of the toothpaste is swallowed, and “(…) a high percentage of children in the two youngest age group [18–30 months] appeared to ingest between 80 and 100% of the fluoride dispensed. (…)” [24]. At this young age children are at very high risk of developing dental fluorosis [25, 26].

Also, the flavor of the children’s toothpaste has an influence on the intake of fluoride through fluoride toothpastes, i.e., the amount of ingested toothpaste with special flavors has been shown to be higher than those toothpaste with a ‘regular’ flavor [27].

Creeth et al. showed that toothpaste for 3–6-year-old children is overdosed by parents in different countries [28]. In Germany, for example, toothpaste is overdosed by the factor of approximately 4.6, i.e., in a real-life scenario, the dose of a pea-size amount of toothpaste for children does not seem to be feasible.

Thornton-Evan et al. showed that 38.4% of 3–6-year-old children received more than a pea-size amount of toothpaste on their toothbrush in the USA. Out of those, 20.6% used “half-load” of toothpaste and 17.8% even used a “full load” of toothpaste [29]. This is in line with a study by Huebner et al. who showed that most parents in the USA used more toothpaste than recommended for their children and, interestingly, that verbal instructions to limit the toothpaste amount to the recommended dose were not sufficient [30]. Martin et al. found that 26.7% of 45 parents did not dose a smear-size amount of toothpaste for their 21-months-old children (mean age) [31], and Tay et al. found that 47.8% dosed more than a pea-size amount of toothpaste for their 5–6 year-old-children [32].

The dose of an even smaller toothpaste amount, i.e., a grain of rice size-amount of toothpaste for children aged up to 24 months seems to be even more challenging than the dose of a pea-size amount of toothpaste. To test this hypothesis, the aim of this study was to test the real-life dose of fluoride toothpaste by parents for their children aged up to 24 months in Germany.

## Materials and methods

This study was performed from October 11 to 26, 2023. Parents at 5 different daycare centers in Braunschweig, Lower Saxony, Germany, were asked to one-time dose two commercially available fluoride toothpastes for children as they would do normally for their children at home (one dose for each toothpaste). The inclusion criterion was a regular usage of a fluoride toothpaste for the child aged up to 24 months. To increase the number of participants, also parents with children aged > 24 months were included (in this case parents were asked to dose the amount of fluoride toothpaste exactly how they did it when their child was up to 24 months old). The participation in the study was voluntary and only parents who gave their oral consent to dose the test toothpastes and to fill out a questionnaire were included. Please note that after dosing the test toothpastes no tooth brushing was performed.

The commercially available tested toothpastes contain 1000 ppm fluoride and are specially formulated for children from 0 to 6 years. The compositions of the tested toothpastes according to the *International Nomenclature of Cosmetic Ingredients* (INCI) are presented in Table 3.

**Table 3:** Overview of the two commercially available fluoride toothpastes for children used in this study.

Code in the manuscript	Toothpaste name and company	Toothpaste composition	Type of fluoride and concentration
Toothpaste A	Signal Kids Zahnpasta (Unilever, Hamburg, Germany)	Aqua, Hydrogenated Starch Hydrolysate, Hydrated Silica, Aroma, Cellulose Gum, Decyl Glucoside, Sodium Saccharin, Sodium Fluoride, CI 42090.	Sodium fluoride (1000 ppm fluoride)
Toothpaste B	Odol-med 3 Milchzahn Zahnpasta (GSK Consumer Healthcare, München, Germany)	Aqua, Hydrated Silica, Sorbitol, Glycerin, PEG-6, Xanthan Gum, Titanium Dioxide, Aroma, Sodium Saccharin, Sodium Methyl Cocoyl Taurate, Cocamidopropyl Betaine, Sodium Fluoride.	Sodium fluoride (1000 ppm fluoride)

Both test toothpastes were used in original tubes. Parents with subject numbers 001, 003, 005 etc. started dosing toothpaste A (and afterwards toothpaste B), parents with subject numbers 002, 004, 006 etc. started dosing toothpaste B (and afterwards toothpaste A).

The densities of the toothpastes A and toothpaste B were determined by dosing exactly 5 mL toothpaste in a graduated pipette tip (epT.I.P.S. standard, Eppendorf SE, Hamburg, Germany) and weighing the toothpastes. The mean densities including standard deviations of three measures were calculated.

Toothpastes A and B were dosed one-time by the parents on a commercially available children’s toothbrush (Signal toothbrush for children aged 0-6 years; Unilever, Hamburg, Germany), and the amount of toothpaste was weighed. The weight of every toothpaste dose was determined by the following equation:

Absolute application dose (g) = [weight of unused toothbrush (g) + applied toothpaste amount (g)] – weight of unused toothbrush (g).

All toothbrushes were weighed at the study site right before the arrival of the participants because their weight slightly differed (although the same toothbrush type was used). Means, standard deviations, and medians of the toothpaste doses were calculated from all participants of the study per toothpaste group.

An ‘optimum’ grain of rice-size of each fluoride toothpaste (as recommended for children’s toothpaste with 1000 ppm fluoride [6, 7]) was dosed 5 times in a row by an experienced dentists (H.S.) using a natural grain of rice as model and mean, standard deviation, and median were calculated from 5 individual measurements. This toothpaste amount was used as reference in this study.

Additionally, parents were asked about the total frequency of tooth brushing of their children with fluoride toothpaste per day (including tooth brushing at daycare center), the knowledge of conditions of use and warnings for children in relation to fluoride toothpastes with 1000 ppm fluoride [5–7], and the usage of fluoride tablets with vitamin D. All calculations of mean, standard deviation, and median as well as the significance tests (two-sided t-tests) were performed with Microsoft Excel.

## Results

In total, 61 parents participated in this study. The current age of the children was 24 ± 7 months with a range of 10–34 months (median: 24 months). Please note that parents of children aged > 24 months were also included in this study. In this case parents were asked to dose the amount of fluoride toothpaste exactly how they did it when their child was up to 24 months old.

**Fig. 1:**
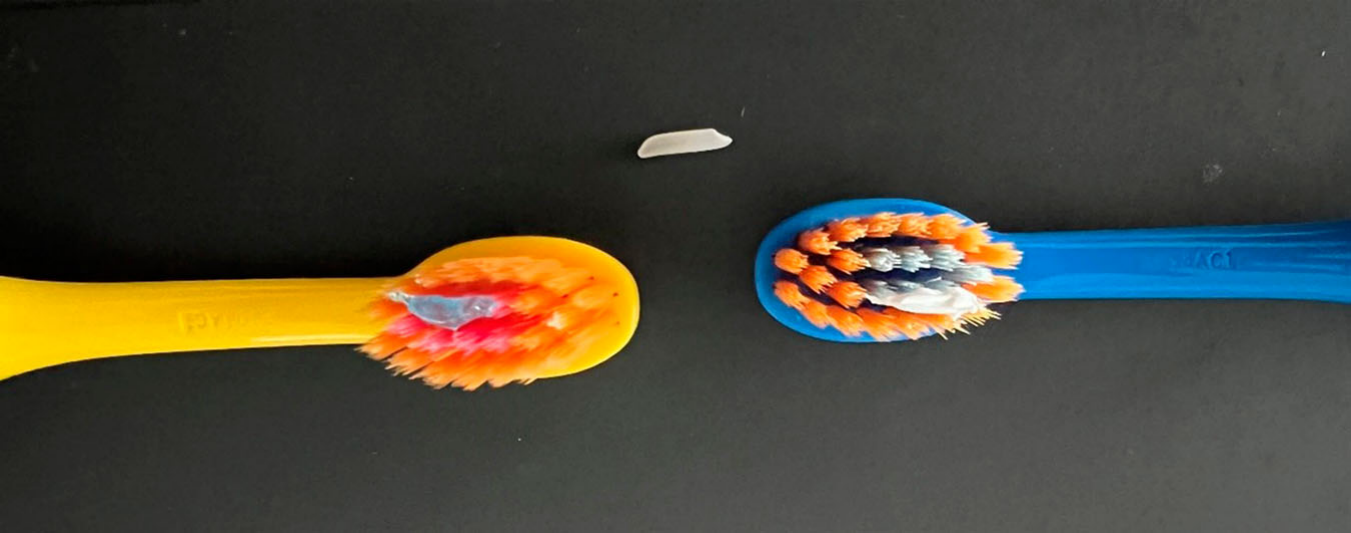
Reference doses. Photograph of the reference doses (i.e., a grain of rice-size amount of toothpaste as recommended for toothpastes for children aged up to 24 months with 1000 ppm fluoride [6, 7]) of toothpaste A (left) and toothpaste B (right) on children’s toothbrushes. A natural grain of rice was used as model. Both amounts were dosed by an experienced dentist (see Table 4 for weight results).

The reference doses of both test toothpastes as recommended (i.e., grain of rice-size of toothpastes) are shown in Fig. 1.

The densities of the tested toothpastes were approximately 1.3 g/mL, i.e., 1.308 ± 0.010 g/mL (toothpaste A) and 1.295 ± 0.012 g/mL (toothpaste B).

The results of this study clearly show that both fluoride toothpastes A and B were overdosed by parents in comparison to the reference dose of a rice-size amount of fluoride toothpaste (according to current guidelines [6, 7]) (Tables 4 and 5). The head-to-head comparison of the reference doses of toothpaste A and toothpaste B to the amounts dosed by parents showed a statistically significant difference (*p* < 0.001). The difference between the parents’ dose of toothpaste A and toothpaste B was not statistically significant (*p* > 0.1).

**Table 4:** Overview of the dose of two commercially available children’s toothpastes with 1000 ppm fluoride by parents in comparison to the reference dose for children aged up to 24 months (grain of rice-size [6, 7]).

	Parents’ dose		Reference dose (dosed by an experienced dentist)	
	Toothpaste A	Toothpaste B	Toothpaste A	Toothpaste B
Number of subjects (numbers of applications)	*N* = 61 [1x]		*N* = 1 [5x]	
Mean dose ± standard deviation (g)	0.263 ± 0.172	0.281 ± 0.145	0.045 ± 0.006	0.039 ± 0.012
Median dose (g)	0.234	0.280	0.042	0.034

**Table 5:** Calculation of the overdosing of the tested fluoride toothpastes by parents in comparison to the reference dose for children aged up to 24 months (grain of rice-size), see also Table 4.

	Factor of overdose of toothpaste A	Factor of overdose of toothpaste B
Mean dose (subjects) (g)/mean dose (reference) (g)	5.9	7.2
Median dose (subjects) (g)/median dose (reference) (g)	5.6	8.2

Figure 3 shows the dosing of toothpaste A and toothpaste B as performed by parents.

Parents answered that their children’s teeth were mainly brushed 2-times a day (62.2%) or 3-times a day (22.9%) (Table 6).

**Table 6:** Frequency of tooth brushing of children aged up to 24 months with fluoride toothpaste per day, including tooth brushing at daycare centers (for calculation of fluoride intake through the tested fluoride toothpastes see Tables 7 and 8).

Frequency of tooth brushing per day	Number	Proportion (%)
1	7	11.4
2	38	62.2
3	14	22.9
4	1	1.6
5	0	0
6	1	1.6

The calculation of the hypothetical fluoride intake through the tested toothpastes is presented in Tables 7 and 8.

**Table 7:** Calculation of fluoride intake through the tested fluoride toothpastes only; assuming 2-times a day tooth brushing with a toothpaste with 1000 ppm (0.10%) fluoride (based on the mean dose in this study; Table 4). Calculation for a 1-year-old child (estimated weight of 9 kg [55]), assuming swallowing of 100% of the fluoride toothpaste [24]. For the correlation between fluoride intake and fluorosis prevalence see Table 11.

	Applied amount of toothpaste (100% absorption) (mg/application)	2x daily application (mg/d)	Retention (1 = 100% swallowing of toothpaste)	Fluoride concentration of the toothpaste (%)	Fluoride intake (mg/d)	Body weight (kg)	Fluoride intake through fluoride toothpaste only (mg/kg bw/d)
**Toothpaste A** Mean dose of toothpaste by parents	263	526	1	0.10	0.526	9	0.058 (> 0.04, i.e., a significantly elevated risk for fluorosis [25])
Toothpaste A Reference dose of grain of rice-size of toothpaste	45	90	1	0.10	0.09	9	0.01
**Toothpaste B** Mean dose of toothpaste by parents	281	562	1	0.10	0.56	9	0.062 (> 0.04, i.e., a significantly elevated risk for fluorosis [25])
**Toothpaste B** Reference dose of grain of rice-size of toothpaste	39	78	1	0.10	0.078	9	0.008

**Table 8:** Calculation of fluoride intake through the tested fluoride toothpastes only; assuming a 3-times a day tooth brushing with a toothpaste with 1000 ppm (0.10%) fluoride (based on the mean dose in this study; Table 4). Calculation for a 1-year-old child (estimated weight of 9 kg [55]), assuming swallowing of 100% of the fluoride toothpaste [24]. For the correlation between fluoride intake and fluorosis prevalence see Table 11.

	Applied amount of toothpaste (100% absorption) (mg / application)	3x daily application (mg/d)	Retention (1 = 100% swallowing of toothpaste)	Fluoride concentration of the toothpaste (%)	Fluoride intake (mg/d)	Body weight (kg)	Fluoride intake through fluoride toothpaste only (mg/kg bw/d)
**Toothpaste A** Mean dose of toothpaste by parents	263	789	1	0.10	0.789	9	0.087 (> 0.04, i.e., a significantly elevated risk for fluorosis [25])
**Toothpaste A** Reference dose of grain of rice-size of toothpaste	45	135	1	0.10	0.135	9	0.015
**Toothpaste B** Mean dose of toothpaste by parents	281	843	1	0.10	0.843	9	0.093 (> 0.04, i.e., a significantly elevated risk for fluorosis [25])
**Toothpaste B** Reference dose of grain of rice-size of toothpaste	39	117	1	0.10	0.117	9	0.013

When asked about the knowledge about the conditions of use and warnings in relation to fluoride toothpastes with 1000 ppm fluoride for children [5–7], 60.6% of the parents answered that they know them and 39.3% do not know them (Table 9).

**Table 9:** Knowledge of parents about conditions of use and warnings in relation to fluoride toothpastes with 1000 ppm fluoride for children (for details see also [5–7]).

Knowledge of conditions of use and warnings in relation to fluoride toothpastes with 1000 ppm fluoride for children	Number	Proportion (%)
Knowledge	Total: 37 - Age recommendation: 28 - Fluoride concentration: 7 - Age recommendation and fluoride concentration: 2	Total: 60.6 - Age recommendation: 45.9 - Fluoride concentration: 11.4 - Age recommendation and fluoride concentration: 3.2
No knowledge	Total: 24	Total: 39.3

Finally, parents were asked about the use of fluoride tablets for their children in combination with fluoride toothpaste. 14.7% used fluoride tablets for their child and 85.2% did not use them (Table 10).

**Table 10:** Regular usage of fluoride tablets with vitamin D for children.

Regular usage of fluoride tablets (in combination with vitamin D)	Number	Proportion (%)
Regular use	Total: 9 - Until 9 months: 1 - Until 12 months: 3 - Until 18 months: 1 - Until 24 months: 2 - No age given: 2	Total: 14.7
No use	Total: 52	Total: 85.2

## Discussion

### Discussion of study results

**Fig. 2:**
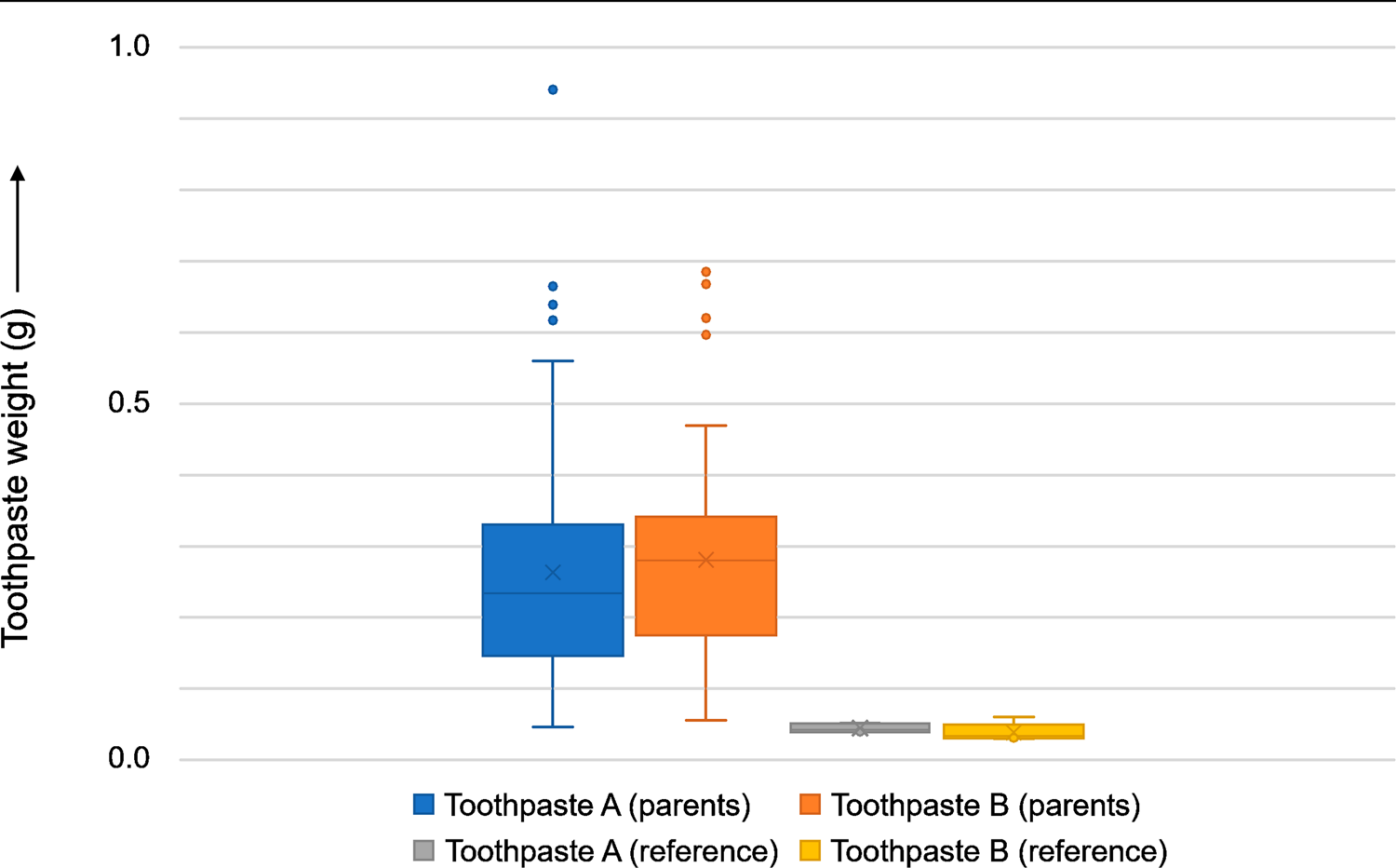
Main study results. Boxplots showing the main study results regarding toothpaste dose (for details see also Table 4).

**Fig. 3:**
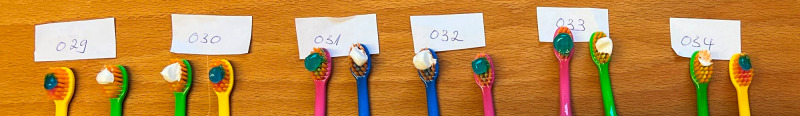
Examples of toothpaste dose. Photograph of examples of toothpaste dose by parents when asked to dose the amount of fluoride toothpaste they usually dose for their child aged up to 24 months at home (blue toothpaste color: toothpaste A; white toothpaste color: toothpaste B).

This study shows that the two tested commercially available fluoride toothpastes for children aged up to 24 months were significantly overdosed by parents: Fluoride toothpaste A was overdosed by a factor 5.9 and fluoride toothpaste B was overdosed by a factor 7.2 (Tables 4, 5 and Figs. 2, 3). This is in line with other studies with older children who should use a pea-size amount of toothpaste but overdosed [28, 29]. Huebner et al. found that parents dosed 0.36 ± 0.26 g toothpaste for 12-35-months-old children, 0.28 ± 0.19 g for 36–59-months-old children, and 0.38 ± 0.26 g for 60–71-months-old-children when asked to dose the amount of toothpaste they usually do at home [30]. Martin et al. found that 26.7% of the parents did not dose a smear-size amount of toothpaste for their 21-months-old children (mean age) [31]. These findings are relevant for the risk assessment of fluoride since e.g., Naccache et al. stated that “(…) the quantity of dentifrice used was the most important factor affecting the ingestion of fluoride through toothbrushing by young children.” [33]

It is challenging to calculate the overall fluoride intake for children up to 24 months because of the various sources of fluoride including food, supplements, drinking water etc. [15–17]. Moreover, country-specific aspects of fluoride exposure such as artificially fluoridated drinking water, use of fluoridated salt etc. must be considered. However, just taking into account the dose of fluoride toothpaste in this study, the fluoride intake exceeds the ‘upper limit’ of fluoride to avoid the development of dental fluorosis [25]; see Tables 7 and 8 for calculations using the example of a 12-months-old child. For these calculations a 2-times and a 3-times daily application of the toothpaste were assumed (Table 6). Hong et al. studied the correlation between fluoride intake and fluorosis and found that “(…) Average daily intake of 0.04–0.06 mg F/kg/bw showed a significantly elevated risk for fluorosis (23.0% for maxillary central incisors, 14.5% for first molars), while fluorosis risk was even higher for average intake above 0.06 mg F/kg/bw (38.0% for maxillary central incisors, 32.4% for first molars). (…)” [25] (see Table 11 for details).

**Table 11:** Overview on the fluorosis prevalence in dependence on different fluoride intake periods (fluorosis on both permanent maxillary central incisors, taken from [25]).

Fluoride intake period (months)	<0.04 mg F/kg/day	0.04–0.06 mg F/kg/day	>0.06 mg F/kg/day
	Prevalence of fluorosis (%)		
0–24	15.7	25.4	32.7
12–24	16.3	27.8	32.5

For a comprehensive risk assessment, fluoride intake also from other sources should be added. This has not been performed in this study because of the complexity of various fluoride sources, see above. However, the fluorosis risk will further increase when all possible additional fluoride sources will be added to the fluoride exposure from fluoride toothpaste. Moreover, and based on recent in vivo studies, dental fluorosis should not be the only endpoint to perform a comprehensive risk assessment of fluoride but other endpoints such as neurotoxicity should be also included in future studies [10, 14]. It has been clearly stated that when parents use a fluoride toothpaste for the oral care of their children that: “The toothpaste should be applied by parents in correct dose to reliably avoid excessive intake.” [7] However, as shown in this study, parents included in our study did not dose the correct amount of fluoride toothpaste as recommended [6, 7], but significantly overdosed.

In the field of toothpastes it is important to emphasize that, in contrast to adults, children up to 24 months swallow most or all of the toothpaste [24], i.e., the use of fluoride toothpaste at this age must be seen as systemic fluoride exposure.

It is important to note that two commonly used toothpastes with 1000 ppm fluoride specifically formulated for children from the first tooth on were used in original toothpaste with the original diameter of the opening was used. Thus, the resulted presented here are representative of how parents dose fluoride toothpaste for their children at home.

In the scientific literature there have been attempts described to limit the dose of fluoride children’s toothpaste (e.g., by using a dispensing device [34]), however, this has not been established on the market. Additionally, verbal instructions do not seem to be useful as Hubner et al. conclude that: “(…) Most parents use more fluoridated toothpaste than is recommended for young children and verbal instructions to limit the dose are ineffective. (…).” [30] Furthermore, the excess doses sized of toothpastes in TV commercials are, besides favorable flavor for children [27], likely to be another reason why young children are overdosed with respect to the amount of toothpaste place on their toothbrushes [32]. An interesting study in this field was published by Basch et al. on the advertisement of children’s toothpaste in parenting magazines in the US [35]. They found that “(…) Of the 31 advertisements that depicted a picture of a toothbrush with toothpaste, all but one (96.8%) depicted a full swirl of toothpaste covering the entire toothbrush head, which is well over the recommended amount. (…)” [35].

There are papers stating that there are fixed-values for the weight of a grain of rice-size amount of toothpaste (i.e., 0.125 g) and a pea-size amount of toothpaste (i.e., 0.25 g) [6]. However, we found that an ‘optimal’ dose of a rice size-amount of toothpaste was even smaller than 0.125 g for the tested toothpastes (in our study: fluoride toothpaste A: 0.045 ± 0.006 g; fluoride toothpaste B: 0.039 ± 0.012) (Table 4). This may be explained by different toothpaste formulations which may lead to different densities of toothpastes. However, the density of the tested toothpastes described in the dose study by Creeth et al. was almost identical to the density of the tested toothpastes in our study (i.e., around 1.3 g/mL) [28]. Since the label text on toothpaste tubes for children with 1000 ppm fluoride in the Germany recommends grain of rice-size amounts of toothpaste for children up to 24 months (and not the dose of a weight of 0.125 g toothpaste) we used the reference weights determined with an ‘optimal’ grain of rice-dose of the toothpastes.

It is important to mention that a notable proportion of parents (39.3%) in our study were not aware about the special conditions of use and warnings in relation to fluoride toothpastes with 1000 ppm fluoride for children [5–7] (Table 9). In future studies it should be determined if another presentation of the label text (e.g., with special graphics and/or enlarged text) would increase the awareness of the special conditions of use and warnings mandatory for toothpastes with 1000 ppm fluoride for children. In a study by Chen et al. 66% of parents were not aware of the special recommendations of dose for fluoride toothpaste for children [36]. Moreover, in this published study, even if the parents were familiar with the guidelines, they, nevertheless, over dispensed a smear-size amount of toothpaste, i.e., the mean 0.21 g but it should have been 0.09 g, as well as they over dispensed a pea-size amount of toothpaste, i.e., the mean was 0.44 g but it should have been 0.22 g [36].

Finally, it is important to note that there are some parents (14.7%) who used not only fluoride toothpaste for their children up to 24 months but also fluoride tablets (Table 10), which can further increase to risk of developing fluorosis.

This study has some limitations which are described below. A limitation of this study is that only two children’s toothpaste and one toothbrush were tested. Thus, a future study could include also other toothpaste and toothbrush brands. Moreover, studies with more participants from different regions in Germany (as well as from other countries) and with a more detailed analysis, e.g., on gender and age as well as on the socioeconomic background of the parents, could be performed. Parents were asked one-time to dose the toothpastes, thus, future studies could analyze the dosing behavior over a longer period.

A strength of our study is that parents were asked to dose the amount they dose at home for their children and not to dose an amount that was shown to them, i.e., this study tested the real-life scenario.

### Fluoride-free toothpastes for children aged up to 24 months

Taken together, the results of this study show that correct dose of a grain of rice size amount of fluoride toothpaste was not dispensed. Thus, to reduce the overall fluoride intake and to prevent the development of dental fluorosis and to avoid other side effects associated with fluoride, fluoride-free toothpaste could be a viable option for the oral care of infants and toddlers. It is important to emphasize that fluoride-free toothpastes should include an anti-caries agent [2].

With a rice-size amount of fluoridated toothpaste, it is not known if such a drastic reduction in the amount of fluoride in a single dose would still be anti-cariogenic. There is at least one clinical trial where a grain of rice-size fluoride toothpaste was tested and it seem to still be effective [37].

Different fluoride-free active ingredients based on calcium phosphates have been described in oral care, e.g., hydroxyapatite, casein phosphopeptide-amorphous calcium phosphate, calcium sodium phosphosilicate, and β-tricalcium phosphate [38]. Out of those fluoride alternatives, hydroxyapatite has been studied most [38].

The clinical efficacy of hydroxyapatite toothpastes in caries protection has been clinically demonstrated [39–45]. This active ingredient mimics the human enamel crystallites [46], and is safe if accidently swallowed and does not pose a fluorosis-risk [47], i.e., it is ideally suited for the oral care of infants and toddlers [48]. Hydroxyapatite has been shown to remineralize early caries in human primary teeth and to prevent demineralization under in situ conditions [43]. Additionally, hydroxyapatite reduces the bacterial colonization to tooth surfaces without having biocide properties [49].

A general advantage of fluoride-free toothpastes for children is that they can be dosed in higher amounts (i.e., the dose is not limited to grain of rice-size or pea-size amounts) which significantly increase the cleaning efficacy of the toothpaste (Table 12) [50].

**Table 12:** Cleaning efficacy of different toothpaste amounts after 120 s. brushing in vitro, taken from [50].

Toothpaste amount	Maximum full length of brush	Minimum full length of brush	Pea-size	Grain of rice-size
Cleaning efficacy (%)	77.4 ± 5.0	75.7 ± 3.4	54.1 ± 6.7	48.2 ± 7.1

## Conclusions

According to current guidelines, toothpastes with 1000 ppm fluoride for children aged up to 24 months should be dosed as a grain of rice-size to limit the overall fluoride intake. However, in this study, parents significantly overdosed the two tested fluoride toothpastes by the factor of 5.9 (toothpaste A) and 7.2 (toothpaste B) compared to the reference dose, respectively. This is in line with other published studies on toothpaste dose. Children up to age 24 months swallow most or all of the toothpaste. This is a matter of concern since an overdose of fluoride can contribute to a higher risk for the development of chronic side effects, e.g., the development of dental fluorosis and other side effects associated with fluoride, especially at young age. Moreover, it is critical to mention that, although regularly using fluoride toothpaste for their children, 39.3% of parents in this study had no knowledge of conditions of use and warnings in relation to fluoride toothpastes with 1000 ppm fluoride.

This is the first study which have quantitatively analyzed how much fluoride toothpaste is dosed by parents in Germany for their children aged up to 24 months. A key finding of this study is that analyzing the fluoride intake from the fluoride toothpaste only (calculated from the mean toothpaste doses), the limit of the fluoride intake to prevent dental fluorosis is exceeded. Fluoride from other sources (fluoridated water, fluoride tablets, fluoridated salt etc.) will further increase this fluorosis risk.

Additionally, our study, supported by other studies, questions the recommended size of a grain of rice-size amount of fluoride toothpaste since the practical implementation seems to be not likely under real-life conditions.

Since some fluoride sources e.g., from food or natural water cannot be avoided by parents, at least they can switch to a fluoride-free toothpaste for the oral care of their infants and toddlers. There are safe and efficient alternatives to fluoride toothpastes, e.g., toothpastes with calcium phosphates which can also be dosed in higher amounts (i.e., the use of fluoride-free toothpastes is not limited to a pea-size or a grain of rice-size amounts of toothpaste).

## Data Availability

All relevant data are included in the manuscript.
